# 1,4-Diaza­bicyclo­[2.2.2]octa­ne–*trans*,*trans*-hexa-2,4-dienedioic acid (1/1)

**DOI:** 10.1107/S160053681202942X

**Published:** 2012-07-04

**Authors:** Suk-Hee Moon, Ki-Min Park

**Affiliations:** a Department of Food & Nutrition, Kyungnam College of Information and Technology, Busan 617-701, Republic of Korea; b Department of Chemistry and Research Institute of Natural Sciences, Gyeongsang National University, Jinju 660-701, Republic of Korea

## Abstract

The title 1:1 co-crystal, C_6_H_12_N_2_·C_6_H_6_O_4_, the dicarb­oxy­lic acid mol­ecule is close to planar [r.m.s. deviation from the mean plane = 0.07 (1) Å]. In the crystal, the two mol­ecules are arranged alternately and are linked by O—H⋯N hydrogen bonds, leading to the formation of a chain along the [20-1] direction. The chains are assembled into a two-dimensional framework parallel to the (102) plane through weak C—H⋯O hydrogen bonds between the two types of mol­ecules.

## Related literature

For background to the applications of co-crystals, see: Bhogala & Nangia (2003 ▶); Gao *et al.* (2004 ▶); Hori *et al.* (2009 ▶); Weyna *et al.* (2009 ▶). For a related structure, see: Moon & Park (2012 ▶).
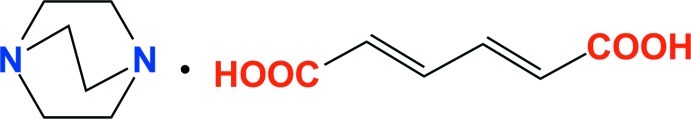


## Experimental

### 

#### Crystal data

C_6_H_12_N_2_·C_6_H_6_O_4_
*M* *_r_* = 254.28
Triclinic, 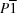

*a* = 8.1547 (2) Å
*b* = 8.9321 (2) Å
*c* = 9.4028 (2) Å
α = 86.258 (1)°
β = 67.376 (1)°
γ = 80.719 (1)°
*V* = 623.90 (2) Å^3^
*Z* = 2
Mo *K*α radiation
μ = 0.10 mm^−1^
*T* = 173 K
0.20 × 0.12 × 0.10 mm

#### Data collection

Bruker APEXII CCD diffractometer
Absorption correction: multi-scan (*SADABS*; Sheldrick, 1996 ▶) *T*_min_ = 0.980, *T*_max_ = 0.990
10893 measured reflections
2719 independent reflections
2382 reflections with *I* > 2σ(*I*)
*R*_int_ = 0.024

#### Refinement

*R*[*F* ^2^ > 2σ(*F* ^2^)] = 0.045
*wR*(*F* ^2^) = 0.125
*S* = 1.06
2719 reflections
163 parameters
H-atom parameters constrained
Δρ_max_ = 0.39 e Å^−3^
Δρ_min_ = −0.37 e Å^−3^

### 

Data collection: *APEX2* (Bruker, 2006 ▶); cell refinement: *SAINT* (Bruker, 2006 ▶); data reduction: *SAINT*; program(s) used to solve structure: *SHELXTL* (Sheldrick, 2008 ▶); program(s) used to refine structure: *SHELXTL*; molecular graphics: *SHELXTL* and *DIAMOND* (Brandenburg, 1998 ▶); software used to prepare material for publication: *SHELXTL*.

## Supplementary Material

Crystal structure: contains datablock(s) I, global. DOI: 10.1107/S160053681202942X/wn2482sup1.cif

Structure factors: contains datablock(s) I. DOI: 10.1107/S160053681202942X/wn2482Isup2.hkl

Supplementary material file. DOI: 10.1107/S160053681202942X/wn2482Isup3.cml

Additional supplementary materials:  crystallographic information; 3D view; checkCIF report

## Figures and Tables

**Table 1:** Hydrogen-bond geometry (Å, °)

*D*—H⋯*A*	*D*—H	H⋯*A*	*D*⋯*A*	*D*—H⋯*A*
O1—H1⋯N1	0.84	1.70	2.5299 (14)	170
O3—H3⋯N2^i^	0.84	1.71	2.5447 (15)	170
C3—H3*A*⋯O2^ii^	0.95	2.53	3.4182 (17)	155
C8—H8*A*⋯O3^ii^	0.99	2.60	3.4255 (18)	141
C9—H9*A*⋯O1^iii^	0.99	2.56	3.0789 (17)	113

Symmetry codes: (i) 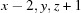; (ii) 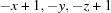; (iii) 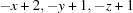.

**Fig. 1.**
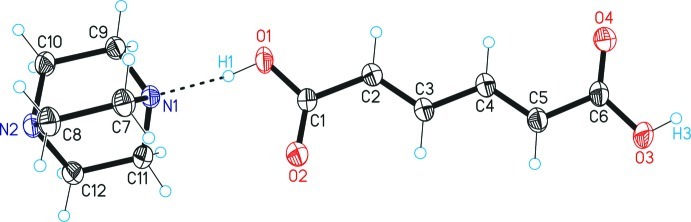
The molecular structure of the title compound, showing displacement ellipsoids drawn at the 50% probability level. Hydrogen atoms are shown as spheres of arbitrary radius. The dashed line indicates a hydrogen bond.

**Fig. 2.**
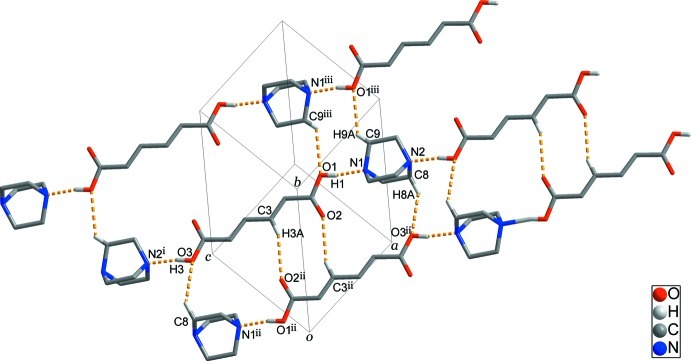
Crystal packing of the title compound with intermolecular O—H···N and C—H···O hydrogen bonds shown as dashed lines. (Symmetry codes: i) x - 2, y, z + 1; ii) -x + 1, -y, -z + 1; iii) -x + 2, -y + 1, -z + 1.) H atoms not involved in the hydrogen bond interactions have been omitted for clarity.

## supplementary crystallographic information

### Comment

Co-crystals made up of two or more components have attracted much attention in recent years owing to their contributions to supramolecular chemistry (Bhogala & Nangia, 2003; Gao *et al.*, 2004), materials chemistry (Hori *et al.*, 2009) and pharmaceutical chemistry (Weyna *et al.*, 2009). As a part of our recent efforts to construct supramolecular architectures using the co-crystal strategy, the crystal structure of a co-crystal consisting of *trans*,*trans*-hexa-2,4-dienedioic acid and 4,4'-bipyridine molecules has been reported by us (Moon & Park, 2012). In this paper we present a co-crystal structure of *trans*,*trans*-hexa-2,4-dienedioic acid with 1,4-diazabicyclo[2.2.2]octane.

The title compound is shown in Fig. 1. The asymmetric unit contains one 1,4-diazabicyclo[2.2.2]octane molecule and one *trans*,*trans*-hexa-2,4-dienedioic acid molecule. The dicarboxylic acid molecule is essentially planar, with an r.m.s. deviation from the mean plane of 0.07 Å.

In the crystal structure, both components are arranged alternately, and linked by intermolecular O—H···N hydrogen bonds, leading to the formation of a one-dimensional chain. Additionally, the chains are assembled into a two-dimensional framework through weak intermolecular C—H···O hydrogen bonds between 1,4-diazabicyclo[2.2.2]octane and dicarboxylic acid molecules (Fig. 2, Table 1).

### Experimental

A mixture of stoichiometric amounts of *trans*,*trans*-hexa-2,4-dienedioic acid and 1,4-Diazabicyclo[2.2.2]octane in DMF (in a 1:1 volume ratio) was heated until the two components dissolved and was then kept at room temperature. Upon slow evaporation of the solvent, *X*–ray quality single crystals were obtained.

### Refinement

All H-atoms were positioned geometrically and refined using a riding model. C—H = 0.95 Å for C*sp*^2^, C—H = 0.99 Å for methylene C and O—H = 0.84 Å for the hydroxyl groups; *U*_iso_(H) = 1.2*U*_eq_(parent atom).

### Crystal data

**Table:**

C_6_H_12_N_2_·C_6_H_6_O_4_	*Z* = 2
*M**_r_* = 254.28	*F*(000) = 272
Triclinic, *P*1	*D*_x_ = 1.354 Mg m^−^^3^
Hall symbol: -P 1	Mo *K*α radiation, λ = 0.71073 Å
*a* = 8.1547 (2) Å	Cell parameters from 5075 reflections
*b* = 8.9321 (2) Å	θ = 3.3–28.4°
*c* = 9.4028 (2) Å	µ = 0.10 mm^−^^1^
α = 86.258 (1)°	*T* = 173 K
β = 67.376 (1)°	Plate, colourless
γ = 80.719 (1)°	0.20 × 0.12 × 0.10 mm
*V* = 623.90 (2) Å^3^	

### Data collection

**Table:**

Bruker APEXII CCD diffractometer	2719 independent reflections
Radiation source: fine-focus sealed tube	2382 reflections with *I* > 2σ(*I*)
Graphite monochromator	*R*_int_ = 0.024
φ and ω scans	θ_max_ = 27.0°, θ_min_ = 2.3°
Absorption correction: multi-scan (*SADABS*; Sheldrick, 1996)	*h* = −10→9
*T*_min_ = 0.980, *T*_max_ = 0.990	*k* = −11→11
10893 measured reflections	*l* = −12→12

### Refinement

**Table:**

Refinement on *F*^2^	Primary atom site location: structure-invariant direct methods
Least-squares matrix: full	Secondary atom site location: difference Fourier map
*R*[*F*^2^ > 2σ(*F*^2^)] = 0.045	Hydrogen site location: inferred from neighbouring sites
*wR*(*F*^2^) = 0.125	H-atom parameters constrained
*S* = 1.06	*w* = 1/[σ^2^(*F*_o_^2^) + (0.0641*P*)^2^ + 0.2432*P*] where *P* = (*F*_o_^2^ + 2*F*_c_^2^)/3
2719 reflections	(Δ/σ)_max_ < 0.001
163 parameters	Δρ_max_ = 0.39 e Å^−^^3^
0 restraints	Δρ_min_ = −0.37 e Å^−^^3^

### Special details

**Table:**

Geometry. All e.s.d.'s (except the e.s.d. in the dihedral angle between two l.s. planes) are estimated using the full covariance matrix. The cell e.s.d.'s are taken into account individually in the estimation of e.s.d.'s in distances, angles and torsion angles; correlations between e.s.d.'s in cell parameters are only used when they are defined by crystal symmetry. An approximate (isotropic) treatment of cell e.s.d.'s is used for estimating e.s.d.'s involving l.s. planes.
Refinement. Refinement of *F*^2^ against ALL reflections. The weighted *R*-factor *wR* and goodness of fit *S* are based on *F*^2^, conventional *R*-factors *R* are based on *F*, with *F* set to zero for negative *F*^2^. The threshold expression of *F*^2^ > σ(*F*^2^) is used only for calculating *R*-factors(gt) *etc*. and is not relevant to the choice of reflections for refinement. *R*-factors based on *F*^2^ are statistically about twice as large as those based on *F*, and *R*- factors based on ALL data will be even larger.

### Fractional atomic coordinates and isotropic or equivalent isotropic displacement parameters (Å^2^)

**Table:**

	*x*	*y*	*z*	*U*_iso_*/*U*_eq_	
O1	0.76168 (14)	0.35311 (12)	0.48540 (14)	0.0383 (3)	
H1	0.8660	0.3185	0.4255	0.046*	
O2	0.70091 (14)	0.13027 (12)	0.44151 (14)	0.0356 (3)	
O3	−0.25828 (14)	0.13485 (13)	0.96677 (14)	0.0384 (3)	
H3	−0.3611	0.1718	1.0276	0.046*	
O4	−0.20125 (14)	0.37072 (12)	0.96904 (14)	0.0370 (3)	
C1	0.65577 (17)	0.25426 (15)	0.50280 (15)	0.0231 (3)	
C2	0.46734 (18)	0.30803 (16)	0.60830 (16)	0.0253 (3)	
H2	0.4375	0.4100	0.6432	0.030*	
C3	0.33925 (17)	0.22026 (15)	0.65586 (15)	0.0221 (3)	
H3A	0.3679	0.1191	0.6190	0.027*	
C4	0.15787 (17)	0.27194 (16)	0.76141 (15)	0.0234 (3)	
H4	0.1279	0.3750	0.7929	0.028*	
C5	0.03082 (18)	0.18318 (16)	0.81672 (16)	0.0257 (3)	
H5	0.0598	0.0800	0.7857	0.031*	
C6	−0.15544 (18)	0.23910 (16)	0.92551 (16)	0.0250 (3)	
N1	1.08788 (14)	0.27644 (12)	0.31399 (13)	0.0215 (3)	
N2	1.41841 (14)	0.22012 (13)	0.14620 (13)	0.0230 (3)	
C7	1.11006 (18)	0.27117 (17)	0.15051 (16)	0.0261 (3)	
H7A	1.0583	0.3700	0.1199	0.031*	
H7B	1.0455	0.1919	0.1366	0.031*	
C8	1.31022 (19)	0.23589 (17)	0.04914 (16)	0.0279 (3)	
H8A	1.3332	0.1406	−0.0081	0.033*	
H8B	1.3454	0.3187	−0.0267	0.033*	
C9	1.18363 (18)	0.39594 (16)	0.33479 (16)	0.0252 (3)	
H9A	1.1706	0.3982	0.4438	0.030*	
H9B	1.1302	0.4963	0.3087	0.030*	
C10	1.38327 (18)	0.36395 (16)	0.23055 (17)	0.0273 (3)	
H10A	1.4159	0.4484	0.1563	0.033*	
H10B	1.4575	0.3558	0.2936	0.033*	
C11	1.16529 (18)	0.12776 (15)	0.35852 (16)	0.0252 (3)	
H11A	1.1041	0.0467	0.3432	0.030*	
H11B	1.1471	0.1290	0.4688	0.030*	
C12	1.36674 (19)	0.09588 (16)	0.25949 (17)	0.0274 (3)	
H12A	1.4366	0.0877	0.3263	0.033*	
H12B	1.3943	−0.0016	0.2047	0.033*	

### Atomic displacement parameters (Å^2^)

**Table:**

	*U*^11^	*U*^22^	*U*^33^	*U*^12^	*U*^13^	*U*^23^
O1	0.0164 (5)	0.0337 (6)	0.0531 (7)	−0.0076 (4)	0.0034 (5)	−0.0147 (5)
O2	0.0232 (5)	0.0290 (5)	0.0455 (7)	−0.0053 (4)	−0.0009 (5)	−0.0109 (5)
O3	0.0190 (5)	0.0351 (6)	0.0470 (7)	−0.0081 (4)	0.0059 (5)	−0.0094 (5)
O4	0.0240 (5)	0.0287 (6)	0.0456 (7)	−0.0033 (4)	0.0006 (5)	−0.0012 (5)
C1	0.0176 (6)	0.0246 (7)	0.0252 (7)	−0.0034 (5)	−0.0058 (5)	−0.0002 (5)
C2	0.0195 (7)	0.0242 (6)	0.0289 (7)	−0.0022 (5)	−0.0056 (5)	−0.0027 (5)
C3	0.0177 (6)	0.0254 (6)	0.0218 (6)	−0.0017 (5)	−0.0065 (5)	−0.0001 (5)
C4	0.0178 (6)	0.0272 (7)	0.0235 (6)	−0.0019 (5)	−0.0067 (5)	0.0001 (5)
C5	0.0191 (7)	0.0291 (7)	0.0256 (7)	−0.0029 (5)	−0.0047 (5)	−0.0024 (5)
C6	0.0179 (6)	0.0302 (7)	0.0243 (7)	−0.0040 (5)	−0.0052 (5)	0.0016 (5)
N1	0.0163 (5)	0.0238 (6)	0.0222 (6)	−0.0039 (4)	−0.0041 (4)	−0.0029 (4)
N2	0.0168 (5)	0.0262 (6)	0.0222 (6)	−0.0035 (4)	−0.0030 (4)	−0.0017 (4)
C7	0.0216 (7)	0.0325 (7)	0.0252 (7)	−0.0033 (5)	−0.0101 (5)	−0.0015 (5)
C8	0.0245 (7)	0.0358 (8)	0.0206 (6)	−0.0039 (6)	−0.0054 (5)	−0.0026 (5)
C9	0.0201 (6)	0.0252 (7)	0.0273 (7)	−0.0042 (5)	−0.0043 (5)	−0.0066 (5)
C10	0.0197 (6)	0.0287 (7)	0.0320 (7)	−0.0079 (5)	−0.0055 (6)	−0.0052 (6)
C11	0.0218 (7)	0.0260 (7)	0.0246 (7)	−0.0059 (5)	−0.0049 (5)	0.0024 (5)
C12	0.0229 (7)	0.0245 (7)	0.0295 (7)	−0.0007 (5)	−0.0057 (6)	0.0024 (5)

### Geometric parameters (Å, º)

**Table:**

O1—C1	1.2925 (16)	N2—C8	1.4812 (18)
O1—H1	0.8400	N2—C12	1.4815 (18)
O2—C1	1.2177 (17)	N2—C10	1.4836 (17)
O3—C6	1.2944 (17)	C7—C8	1.5324 (18)
O3—H3	0.8400	C7—H7A	0.9900
O4—C6	1.2217 (18)	C7—H7B	0.9900
C1—C2	1.4924 (18)	C8—H8A	0.9900
C2—C3	1.3284 (19)	C8—H8B	0.9900
C2—H2	0.9500	C9—C10	1.5335 (18)
C3—C4	1.4477 (18)	C9—H9A	0.9900
C3—H3A	0.9500	C9—H9B	0.9900
C4—C5	1.3301 (19)	C10—H10A	0.9900
C4—H4	0.9500	C10—H10B	0.9900
C5—C6	1.4931 (18)	C11—C12	1.5342 (18)
C5—H5	0.9500	C11—H11A	0.9900
N1—C7	1.4800 (17)	C11—H11B	0.9900
N1—C11	1.4825 (17)	C12—H12A	0.9900
N1—C9	1.4835 (16)	C12—H12B	0.9900
			
C1—O1—H1	109.5	H7A—C7—H7B	108.2
C6—O3—H3	109.5	N2—C8—C7	109.89 (11)
O2—C1—O1	125.03 (13)	N2—C8—H8A	109.7
O2—C1—C2	122.44 (12)	C7—C8—H8A	109.7
O1—C1—C2	112.53 (12)	N2—C8—H8B	109.7
C3—C2—C1	123.21 (12)	C7—C8—H8B	109.7
C3—C2—H2	118.4	H8A—C8—H8B	108.2
C1—C2—H2	118.4	N1—C9—C10	109.92 (10)
C2—C3—C4	122.93 (13)	N1—C9—H9A	109.7
C2—C3—H3A	118.5	C10—C9—H9A	109.7
C4—C3—H3A	118.5	N1—C9—H9B	109.7
C5—C4—C3	123.67 (13)	C10—C9—H9B	109.7
C5—C4—H4	118.2	H9A—C9—H9B	108.2
C3—C4—H4	118.2	N2—C10—C9	109.32 (10)
C4—C5—C6	122.70 (13)	N2—C10—H10A	109.8
C4—C5—H5	118.7	C9—C10—H10A	109.8
C6—C5—H5	118.7	N2—C10—H10B	109.8
O4—C6—O3	125.06 (13)	C9—C10—H10B	109.8
O4—C6—C5	121.79 (12)	H10A—C10—H10B	108.3
O3—C6—C5	113.15 (12)	N1—C11—C12	109.41 (10)
C7—N1—C11	109.23 (10)	N1—C11—H11A	109.8
C7—N1—C9	109.70 (11)	C12—C11—H11A	109.8
C11—N1—C9	108.99 (10)	N1—C11—H11B	109.8
C8—N2—C12	109.88 (11)	C12—C11—H11B	109.8
C8—N2—C10	109.04 (11)	H11A—C11—H11B	108.2
C12—N2—C10	108.85 (11)	N2—C12—C11	109.83 (11)
N1—C7—C8	109.49 (11)	N2—C12—H12A	109.7
N1—C7—H7A	109.8	C11—C12—H12A	109.7
C8—C7—H7A	109.8	N2—C12—H12B	109.7
N1—C7—H7B	109.8	C11—C12—H12B	109.7
C8—C7—H7B	109.8	H12A—C12—H12B	108.2
			
O2—C1—C2—C3	7.1 (2)	N1—C7—C8—N2	−0.71 (16)
O1—C1—C2—C3	−172.87 (14)	C7—N1—C9—C10	−58.31 (14)
C1—C2—C3—C4	178.33 (12)	C11—N1—C9—C10	61.24 (14)
C2—C3—C4—C5	−176.16 (14)	C8—N2—C10—C9	61.06 (14)
C3—C4—C5—C6	−179.99 (12)	C12—N2—C10—C9	−58.81 (14)
C4—C5—C6—O4	0.4 (2)	N1—C9—C10—N2	−2.11 (16)
C4—C5—C6—O3	−179.44 (13)	C7—N1—C11—C12	61.50 (14)
C11—N1—C7—C8	−59.61 (14)	C9—N1—C11—C12	−58.35 (14)
C9—N1—C7—C8	59.80 (14)	C8—N2—C12—C11	−57.63 (14)
C12—N2—C8—C7	59.59 (14)	C10—N2—C12—C11	61.71 (14)
C10—N2—C8—C7	−59.64 (14)	N1—C11—C12—N2	−2.54 (15)

### Hydrogen-bond geometry (Å, º)

**Table:**

*D*—H···*A*	*D*—H	H···*A*	*D*···*A*	*D*—H···*A*
O1—H1···N1	0.84	1.70	2.5299 (14)	170
O3—H3···N2^i^	0.84	1.71	2.5447 (15)	170
C3—H3*A*···O2^ii^	0.95	2.53	3.4182 (17)	155
C8—H8*A*···O3^ii^	0.99	2.60	3.4255 (18)	141
C9—H9*A*···O1^iii^	0.99	2.56	3.0789 (17)	113

Symmetry codes: (i) *x*−2, *y*, *z*+1; (ii) −*x*+1, −*y*, −*z*+1; (iii) −*x*+2, −*y*+1, −*z*+1.
